# Body satisfaction and sexuality in pregnant and postpartum women

**DOI:** 10.1192/j.eurpsy.2024.772

**Published:** 2024-08-27

**Authors:** S. Bader, M. Aloulou, Z. Zran, A. Abdelmoula, A. Bouaziz, W. Abbes

**Affiliations:** ^1^Psychiatry; ^2^obstetric gynecology, University Hospital of Gabes, Gabes, Tunisia

## Abstract

**Introduction:**

Pregnancy and postpartum is an important life event associated with profound physiognomic and psychosocial changes affecting the female body in all its physiological, psychic and affective reality. It might influence the sexual function in expectant mothers.

**Objectives:**

To investigate the relationship between the body satisfaction and perception and the sexual function among pregnant and postpartum women.

**Methods:**

It was a cross-sectional study established over a period of 3 months from the June 1^st^, 2023 to August 31, 2023. This study focused on a population of pregnant and postpartum women recruited from outpatient consultations and inpatient of the obstetric gynecology department at the university hospital of Gabes. We used a pre-established sheet exploring socio-demographic data, medical and gyneco-obstetric history, the body mass index (BMI) and informations concerning the marital relationship and the woman’s sexual activity. We administered the validated Arabic version of the Arizona Sexual Experiences Scale (ASEX) to assess sexual functioning and we used the body satisfaction and global self-perception questionnaire (QSCPGS) to explore the body satisfaction and perception.

**Results:**

Fifty-eight women were included. The average age was 35.6±5.5 years; they were from an urban origin in 75%. They were pregnant in the first, second and third trimester in (15.6%, 15.6% and 25% respectively). They were in postpartum in 43.8% of cases with a cesarean delivery in 73.3% and breastfeeding in 56%. All women reported being on good terms with their spouses and satisfied with their sexuality. The usual frequency of sexual relations was (1/day: 22.6%, 1/week: 74.2%, 1/month: 3.2%) and 25% reported wanting to reduce the frequency. The mean ASEX score was 13 ± 4.3 and 47% of the sample had sexual dysfunction. For the total score of the QSCPGS, we observe a mean value of 33 ±28.3, which means that our sample has a good level of positive body satisfaction and self-perception. The mean value of the “body satisfaction” factor is higher (23.7 ± 10.4) than the mean value of the “self-perception” factor (11.4 ± 14.3). The mean value of BMI was 28.74 ± 4.4 wich means an overweigh. We found a significant association between the “body satisfaction” factor and the sexual dysfunction (p=0.03), insufficient lubrication (p=0.01) and difficulty reaching orgasm (p=0.001).

**Conclusions:**

We found that body and physical changes among pregnant and postpartum women can negatively affects their body perception and it might deteriorate its global sexual function. Further researches are recommended to study other potential factors affecting sexual function during this period.

**Disclosure of Interest:**

None Declared

